# Gut microbial signatures associated with the Indian lean MASLD phenotype

**DOI:** 10.3389/fnut.2025.1673517

**Published:** 2025-09-24

**Authors:** Priyankar Dey

**Affiliations:** Department of Biotechnology, Thapar Institute of Engineering and Technology, Patiala, Punjab, India

**Keywords:** NAFLD, NASH, MASLD, microbiota, lean, obese, BMI

## Abstract

Lean Metabolic Dysfunction-associated Steatotic Liver Disease (MASLD) is a substantial challenge in India, manifesting in individuals with normal BMI and indicative of a *‘metabolically obese normal weight*’ phenotype. This review delineates the unique gut microbial signatures that characterize Indian lean MASLD, distinguishing it from obese MASLD. Principal modifications encompass substantial decreases in *Faecalibacterium* (particularly *F. prausnitzii*), *Ruminococcus*, *Lactobacillus*, *Lachnospira*, and *Subdoligranulum*, alongside an increase in pro-inflammatory *Escherichia-Shigella*. Dysbiotic patterns in India are influenced by factors such as fiber-deficient diets rich in refined carbohydrates, visceral obesity, insulin resistance, and genetic predispositions, including the PNPLA3 rs738409 polymorphism. Microbial alterations can contribute to disease by compromising gut barrier integrity, facilitating endotoxemia, and affecting the generation of beneficial metabolites. The combination signature of increased *Escherichia-Shigella* and decreased *Lachnospira/Subdoligranulum* exhibits significant diagnostic accuracy for detecting lean MASLD in the Indian population. These findings highlight lean MASLD as a mechanistically unique condition necessitating customized diagnostic and treatment approaches beyond standard weight management.

## 1 Introduction

Non-alcoholic Fatty Liver Disease (NAFLD) encompasses a wide range of hepatic disorders, beginning with simple hepatic steatosis (non-alcoholic fatty liver; NAFL), characterized by the buildup of lipids in the liver. This may evolve to Non-alcoholic Steatohepatitis (NASH), a more severe variant marked by inflammation and hepatocellular damage, frequently resulting in advanced liver conditions such as fibrosis, cirrhosis, and hepatocellular carcinoma (HCC) (1). The terminology related to fatty liver disease has recently evolved, introducing the unifying terms Metabolic Dysfunction-associated Steatotic Liver Disease (MASLD) (2) and Metabolic Dysfunction-associated Steatohepatitis (MASH) (3). This reclassification transcends the prior focus on alcohol use, clearly recognizing the robust metabolic processes of the condition (4). The diagnosis of MASLD necessitates evidence of hepatic steatosis, characterized by above 5% fat accumulation in hepatocytes, together with the presence of at least one of five cardiometabolic risk factors. The criteria encompass overweight or obesity, prediabetes or diabetes mellitus, raised triglycerides, and low high-density lipoprotein levels (4). This change in terminology and diagnostic standards expands the clinical viewpoint, promoting a multidisciplinary approach for patient management. The extensive clinical scope, especially pertinent in India given the significant prevalence of diabetes and prediabetes, emphasizes the growing necessity for a comprehensive, multidisciplinary approach to diagnosis and management.

The role of gut microbiota in the pathogenesis of MASLD is well established (5, 6). Nevertheless, studying the gut microbiota composition in lean MASLD patients is crucial, as their condition exemplifies a notable metabolic contradiction that contests the traditional obesity-focused concept of hepatic disease causation (7). Given that these patients do not possess the primary contributor to insulin resistance and hepatic steatosis (excess adiposity), the progression of their disease clearly suggests the involvement of alternate, significant pathogenic pathways. Investigating their microbiome could allow understanding the microbial contributions from the overwhelming effects of obesity, potentially identifying unique microbial signatures, pathways (e.g., specific bile acid alterations, endotoxin production, or metabolite generation), or dysbiosis patterns specifically driving liver injury in this phenotype. This knowledge could be essential for comprehending fundamental disease mechanisms beyond adiposity, developing targeted diagnostic biomarkers for lean individuals who are frequently underdiagnosed, and formulating innovative microbiome-modulating therapeutics effective across all NAFLD subtypes, particularly for this high-risk group where conventional weight-loss recommendations are less relevant.

## 2 The emerging phenotype of lean MASLD

Lean MASLD denotes the occurrence of fatty liver disease in persons who do not satisfy the criteria for overweight or obesity, generally characterized by a Body Mass Index (BMI) below 25 kg/m^2^ among Asian populations (8). Notwithstanding their normal BMI, these patients often display modest yet considerable changes in body composition, including increased total body and regional adiposity, especially in visceral and deep subcutaneous adipose tissues. Standard anthropometric measurements frequently fail to appropriately identify concerns related to fat compartmentalization. This phenotype is commonly referred to as *‘metabolically obese normal weight*’ (MONW) (8). Lean MASLD is an escalating global health issue, with an estimated prevalence of 4.1%, and is particularly prevalent among Asian populations (9). In India, the incidence of MASLD in lean individuals is significant, as evidenced by a biopsy-based study indicating that 33.7% of lean liver donors were affected by MASLD (10). The prevalence of MASLD in South Asia varies significantly, ranging from 9 to 45% (11). Research in India indicates that lean MASLD patients may exhibit markedly abnormal metabolic profiles, such as dyslipidemia and impaired glucose metabolism, similar to those found in obese individuals, despite having lower fasting plasma glucose and insulin resistance levels (12). In fact, it was suggested that central obesity is an independent factor influencing advanced fibrosis in lean individuals with MASLD (13). The significant occurrence of lean MASLD in India, despite ostensibly normal BMI, underscores the ‘*metabolically obese normal weight*’ phenotype and it was suggested that MASLD definition is more suitable to lean NAFLD patients than MAFLD (14). This suggests that BMI alone is an inadequate measure of metabolic health, particularly in Asian populations, and that underlying fat distribution, especially visceral adiposity, and hereditary factors are significant contributors to the disease (4). This discovery contests traditional obesity-focused perspectives of MASLD, highlighting the necessity for more refined diagnostic and risk stratification methodologies. In these settings, the gut microbiome may function as a significant diagnostic or therapeutic target, irrespective of manifest obesity.

Despite a rise in the prevalence among the general population, lean MASLD is frequently underdiagnosed in India owing to various factors. The therapeutic focus on obesity as a principal risk factor results in the neglect of cases in individuals with a normal BMI, even though data indicate that lean MASLD accounts for 9.7% of all MASLD cases (15). Secondly, restricted availability of non-invasive diagnostic techniques (e.g., transient elastography) in primary care environments impedes early identification of such conditions in the non-obese individuals (16). Third, asymptomatic progression and clinician unawareness lead to missed diagnosis, especially in lean individuals with metabolic disorders such as diabetes (4). Ultimately, regional variations in healthcare infrastructure and inadequate incorporation of lean-specific screening in guidelines increase underdiagnosis (17, 18). Overcoming these obstacles necessitates comprehensive diagnostic criteria and improved clinician education regarding metabolic disorders beyond obesity.

## 3 Gut microbial signatures in Indian lean MASLD

### 3.1 Distinct microbial profiles

Recent studies repeatedly demonstrate that lean MASLD patients possess distinct gut microbial signatures and display considerably different gut microbial diversity, a metric of microbial community dissimilarity, in comparison to both obese MASLD patients and healthy controls (12). This indicates that the pathogenic mechanisms behind lean MASLD may be fundamentally different from those in obese MASLD (19). In general, MASLD patients may exhibit an elevation in Proteobacteria at the phylum level (20) and changed Firmicutes/Bacteroidetes ratios (21); however, specific microbial alterations are more severe and distinctive in lean individuals. The persistent identification of unique gut microbial signatures in lean MASLD patients, in contrast to obese MASLD patients, strongly substantiates the argument that lean MASLD is not simply a less severe variant of the obese condition, but rather a distinct mechanistic phenotype (9). This indicates that diagnostic indicators and therapy techniques designed for obese MASLD may not be immediately applicable or ideally successful for lean persons, requiring customized approaches that address their distinct microbial profiles.

### 3.2 Key microbial taxa

Numerous significant microbial genera and species have been associated with lean MASLD, with particular findings relevant to the Indian population (Table 1).

**Table 1:** Gut microbial signatures in Indian lean MASLD and their clinical significance.

Microbial taxa	Change in lean MASLD	Key associations/mechanisms	Diagnostic/therapeutic relevance
*Faecalibacterium*	↓ Decreased	• Specifically reduced *F. prausnitzii* (key butyrate producer)	• Protective role
		• Loss linked to impaired gut barrier, inflammation	• Potential probiotic target (requires supportive prebiotics/diet)
*Ruminococcus*	↓ Decreased	• Contrasts with *increases* often seen in obese MASLD/fibrosis	• Highlights lean vs. obese distinction
		• Includes beneficial (amylolytic) and harmful (*R. gnavus*) species	• Species-level analysis critical
*Lactobacillus*	↓ Decreased	• More depleted vs. obese MASLD	• Probiotic potential complex (strain-dependent)
		• Paradoxically *increased* in advanced fibrosis	• Depletion may reflect severe gut milieu
		• Strain-specific effects	
*Bifidobacterium*	↓ Decreased	• Reduced in overweight/obese MASLD	• Universal therapeutic target (probiotics) across MASLD phenotypes
		• Linked to endotoxemia	
		• Enhances barrier function and metabolism	
*Lachnospira*	↓ Decreased	• Part of a diagnostic signature combination	• Combined with ↓*Subdoligranulum* and ↑*Escherichia-Shigella* for lean MASLD diagnosis (AUC=0.82)
*Subdoligranulum*	↓ Decreased	• Part of a diagnostic signature combination	• Combined with ↓*Lachnospira* and ↑*Escherichia-Shigella* for lean MASLD diagnosis (AUC=0.82)
*Escherichia-Shigella*	↑ Increased	• Pro-inflammatory	• Combined with ↓*Lachnospira* and ↓*Subdoligranulum* for lean MASLD diagnosis (AUC=0.82)
		• Part of a diagnostic signature combination	
*Blautia*	Context-dependent	• Increased linked to PNPLA3 rs738409 CC genotype	• Role ambiguous (potential driver)
		• Associated with inflammation/fibrosis in some studies	• Highlights host gene-microbe interactions

#### 3.2.1 Faecalibacterium

A reduction of *Faecalibacterium* is associated with the pathophysiology of MASLD as demonstrated in a BMI- and sex-matched study of MASLD patients within a large study cohort (22). This association was irrespective of BMI and adiposity. However, a prospective pilot investigation revealed that lean NASH patients had a 3-fold reduction in the abundance of *Faecalibacterium* compared to control groups (19). Reduced *Faecalibacterium*, particularly its predominant strain *F. prausnitzii*, is noted in overall MASLD participants compared to healthy controls, with further reductions in individuals with more severe fibrosis (stage 3–4) (23). *F. prausnitzii* is a prominent butyrate-producing bacterium (24). Butyrate is recognized for its anti-inflammatory characteristics and its function in preserving gut barrier integrity (25). Preclinical studies suggest that *F. prausnitzii* supplementation enhances glucose homeostasis, inhibits hepatic lipid accumulation, mitigates liver damage and fibrosis, and restores compromised gut barrier function in mouse models of MASLD (26). Nonetheless, one study observed that oral gavage of *F. prausnitzii* did not ameliorate diet-induced steatohepatitis in mice, despite its correlation with MASLD severity in humans (23). The persistent finding of diminished *Faecalibacterium* in lean NASH and more severe MASLD, along with its established function as a beneficial butyrate producer, clearly indicates its protective significance. Although why reduced *Faecalibacterium* levels is observed in Indian lean MASLD patients remains unexplored, it is likely attributable to a fiber-deficient diet (27), coupled with a metabolic milieu marked by pronounced insulin resistance, visceral fat accumulation, and chronic inflammation, despite the absence of overt obesity (28). This establishes a gut milieu unsuitable to *F. prausnitzii*, while its reduction further intensifies gut barrier impairment and inflammation, accelerating the advancement of MASLD. Genetic predisposition and environmental variables, such as antibiotic usage, could also contribute to this dysbiotic profile (29). Moreover, the divergence between human associations and mouse intervention studies underscores the intricacy of microbiome interventions, merely introducing a beneficial bacterium may be insufficient if the gut environment is not favorable for its colonization or if other factors, such as diet and host genetics, diminish its efficacy. This indicates that future therapies may require the integration of probiotics with prebiotics or alternative dietary adjustments to establish a conducive environment for these advantageous bacteria.

#### 3.2.2 Ruminococcus

Indian lean MASLD patients exhibited a 3-fold reduction in the amount of *Ruminococcus* (19). In contrast, certain investigations including general MASLD patients indicated elevated amounts of *Ruminococcus* relative to controls (30). Significantly, the abundance of *Ruminococcus* has been independently correlated with liver fibrosis (*F* ≥ 2) (19). *Ruminococcus gnavus* has been associated with pro-inflammatory markers, inflammation, and fibrosis in patients with MASLD (31). The genus *Ruminococcus* exhibits significant heterogeneity, encompassing both advantageous and potentially harmful species, hence complicating the understanding of its function (6). *R. gnavus* is recognized for its impact on host metabolism and its ability to provoke either pro-inflammatory or anti-inflammatory responses (32). The populations are susceptible to nutritional alterations (6). The conflicting results regarding *Ruminococcus* (reduced in lean NASH, while elevated in general MASLD and correlated with fibrosis) highlight the necessity for species-level differentiation and patient stratification (lean vs. obese, fibrosis stage). This implies that certain *Ruminococcus* species may have contradictory effects on liver health, or their influence is contingent upon context (e.g., affected by food or other concurrent dysbiosis). General analyses at the genus level may obscure significant specific effects, necessitating additional research to investigate species-specific roles and their interactions within the intricate gut ecosystem. Nevertheless, the reduced abundance of *Ruminococcus* in Indian lean MASLD patients is likely attributable to a confluence of dietary and metabolic variables. The common diet characterized by high-processed carbohydrates and low-resistant starch (e.g., refined rice and wheat) deprives amylolytic *Ruminococcus* spp. of their principal fermentable substrates (33). Simultaneously, metabolic dysfunction, marked by visceral fat accumulation, insulin resistance, and systemic inflammation even at reduced BMI, facilitates gut dysbiosis and barrier impairment (34). This milieu promotes pro-inflammatory mucin-degrading *Ruminococcus* strains (e.g., *R. gnavus*), which may temporarily proliferate but ultimately lead to pathogenic alterations, while diminishing beneficial fiber-utilizing species essential for butyrate synthesis and gut health.

#### 3.2.3 Lactobacillus

Supplementation of probiotic *Lactobacillus* has proved to ameliorate diet-induced NASH in animal models (35). Lean MASLD patients demonstrated a higher depletion in *Lactobacillus* relative to overweight and obese NASH patients (19). Liver fibrosis (≥ F2) correlated with a heightened prevalence of *Lactobacilli*, while obese MASLD patients exhibited an enrichment of *Lactobacilli* (19). Patients with general MASLD exhibit elevated levels of *Lactobacillus* in comparison to healthy controls (5). Nevertheless, these diverse associations, numerous *Lactobacillus* strains are acknowledged as probiotics that confer advantageous outcomes in rodent models of MASLD, such as enhancing insulin sensitivity, mitigating inflammation, and decreasing lipogenesis (21). Particular strains such as *L. acidophilus* have shown preventive potential against MASLD-related HCC by generating valeric acid and enhancing gut barrier integrity (36). *L. plantarum* can mitigate redox-induced hepatocellular injury (37), and inflammation associated with MASLD (38). Probiotic treatment with several *Lactobacillus* spp. has demonstrated efficacy in ameliorating hepatic steatosis and diminishing liver inflammation (21). The apparently contradicting results regarding *Lactobacillus* (deficiency in lean MASLD but relatively elevated levels in fibrotic and obese MASLD) further indicate species- or strain-specific effects. Although remains unexplored, this is likely attributable to differing metabolic and nutritional interactions. Lean NASH in Indians is significantly linked to the ‘thin-fat’ phenotype, characterized by elevated visceral adiposity and insulin resistance despite a lower BMI, which induces severe gut inflammation and barrier impairment, resulting in a more adverse microenvironment for commensal lactobacilli compared to certain obese conditions (8, 39). Moreover, dietary elements specific to this population, such as elevated consumption of antimicrobial spices (e.g., turmeric, chili) and widespread intake of refined carbohydrates, may more effectively inhibit *Lactobacillus* than diets linked to obesity-related MASLD. Ironically, some *Lactobacillus* spp. may exacerbate inflammation in metabolic disorders, perhaps resulting in increased suppression within the markedly inflammatory lean MASLD environment (40). Although numerous *Lactobacillus* strains are recognized as probiotics that positively influence liver function, an over-proliferation of specific species or a dysbiosis within the genus may exacerbate disease, particularly in cases of severe fibrosis (41). This suggests that merely enhancing *Lactobacillus* proliferation may not consistently yield advantages, a detailed comprehension of particular strains and their metabolic roles is essential for successful probiotic applications.

#### 3.2.4 Bifidobacterium

Patients with overweight MASLD demonstrated reduced abundance of *Bifidobacterium* (19). Within the wider South Asian population, diminished cecal *Bifidobacterium* levels have been linked to increased endotoxemia, which contributes to diabetes, obesity, and MASLD. *Bifidobacterium* spp. are acknowledged as advantageous probiotics (42). They are recognized for their ability to repair intestinal barrier integrity, diminish endotoxemia, and enhance lipid metabolism and insulin sensitivity. *B. animalis* subsp. Lactis V9 has demonstrated the capacity to lower liver transaminases, diminish TLR4 and TLR9 levels, and mitigate liver inflammation (43). The persistent observation of diminished *Bifidobacterium* in overweight NASH and its correlation with endotoxemia in South Asians underscores its essential function in preserving gut barrier integrity and mitigating systemic inflammation. This underscores that *Bifidobacterium* depletion is a prevalent dysbiotic characteristic among various MASLD phenotypes (lean, overweight, obese) and indicates that *Bifidobacterium*-containing probiotics may serve as a universally advantageous therapeutic approach, especially in the Indian context where its levels are observed to be diminished.

#### 3.2.5 Blautia

In a recent study, *Blautia* exhibited a substantial rise in persons possessing the PNPLA3 rs738409 CC genotype throughout a 4-year duration (44). Several investigations involving general MASLD patients have indicated elevated levels of *Blautia* in comparison to controls (30). Nonetheless, alternative therapies, such as inulin administration, have demonstrated the capacity to down-regulate *Blautia* abundance, indicating a potentially adverse impact in some situations (45). The significance of *Blautia* in obesity and liver disease is seen as controversial, with certain research indicating advantageous effects while others recognize it as a contributing component (46). *Blautia* has been demonstrated to induce liver inflammation and hepatic fibrosis in murine models (47). The conflicting results regarding *Blautia* underscore the intricacy of analyzing microbial alterations. The distinct correlation between *Blautia* elevation and the PNPLA3 CC genotype indicates a gene-microbe interaction that may affect disease progression. This underscores that the influence of a certain genus can be significantly context-dependent, shaped by host genetics, co-existing microbial species, and unique eating habits. It warns against oversimplified categorization of ‘good’ and ‘bad’ (48) and advocates for a more refined comprehension of strain-specific functions and their interactions with host variables.

#### 3.2.6 Other signatures

A study conducted in India comparing lean and non-lean MASLD patients revealed a notable increase of *Escherichia-Shigella* and a reduction of *Lachnospira* and *Subdoligranulum* especially in the lean MASLD subgroup (12). The amalgamation of these bacterial genera showed significant diagnostic precision (AUC of 0.82) in differentiating lean from non-lean MASLD patients (12). The identification of *Escherichia-Shigella* enrichment and *Lachnospira/Subdoligranulum* depletion as distinct signatures for Indian lean MASLD is a significant finding, offering concrete, regionally pertinent microbial targets that may function as diagnostic biomarkers or therapeutic intervention points. The elevated diagnostic precision indicates significant potential for clinical utilization. Finally, gut microbial diversity has emerged as a critical indicator of overall metabolic health, where a depleted diversity is generally indicative of metabolic disease. In fact, patients with MASLD show a marked reduction in the gut microbiota diversity compared to healthy cohorts (49–51). However, whether the gut microbial diversity of lean MASLD patients, especially in the case of the Indian cohort, follows a similar trend of reduction, requires further study.

## 4 Conclusion

**Figure 1:**
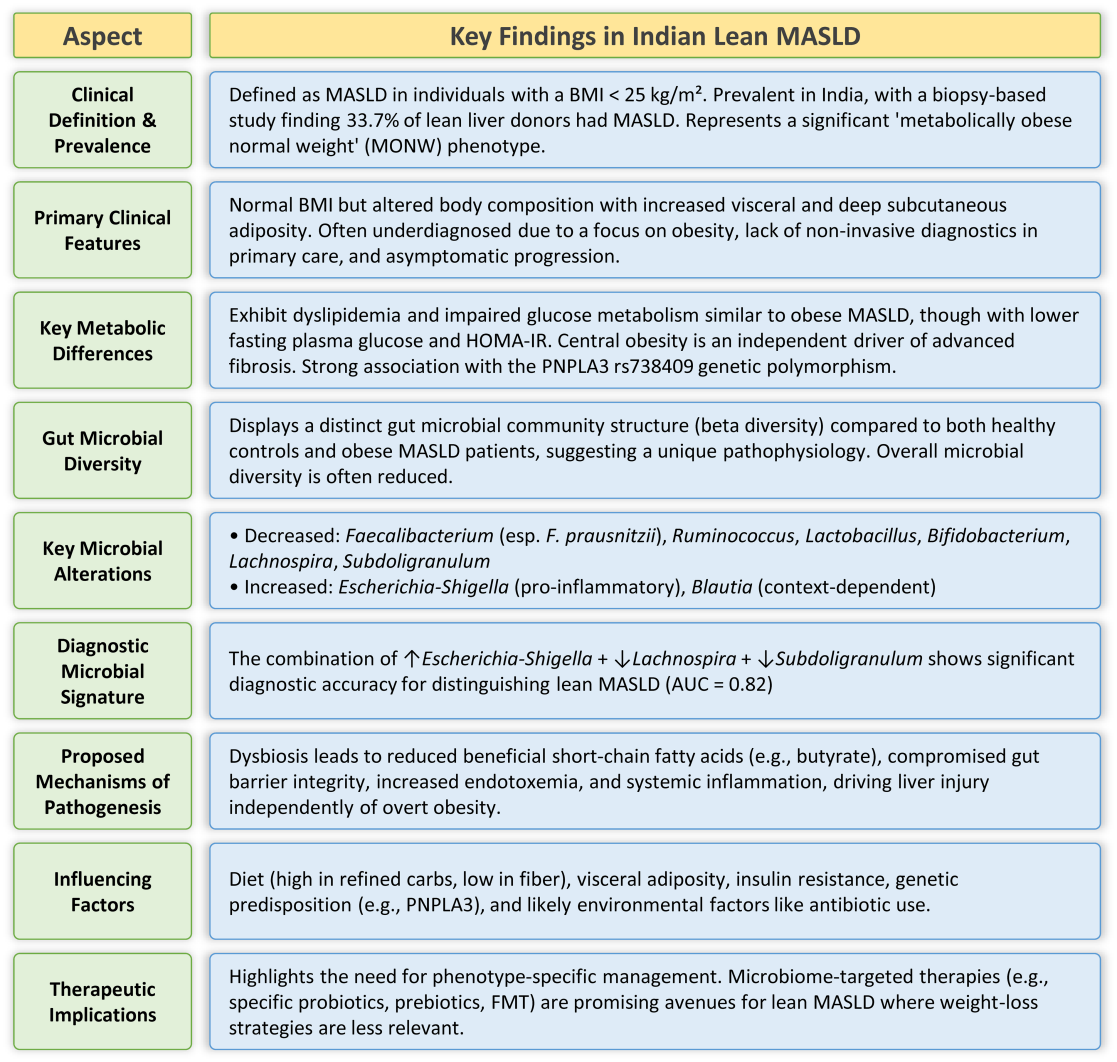
Summary of the major clinical, metabolic, and gut microbial characteristics of the Indian lean MASLD phenotype.

Lean MASLD is a unique and escalating difficulty, especially among the Indian population, as traditional BMI-based diagnoses of obesity may obscure underlying metabolic abnormalities (Figure 1). The pathogenesis of lean MASLD could be closely associated with gut dysbiosis, marked by distinct microbial changes, including diminished *Faecalibacterium*, *Ruminococcus*, and *Lactobacillus* in lean MASLD, as well as a reduction of *Lachnospira* and *Subdoligranulum* and an increase of *Escherichia-Shigella* in Indian lean MASLD. These microbial alterations lead to liver damage by compromising gut barrier integrity, elevating endotoxemia, and modifying the synthesis of microbial metabolites. The advancement of lean MASLD is significantly affected by a complex interaction of genetic factors, particularly the PNPLA3 rs738409 (G/G) genotype, which can directly change the composition of the gut microbiome. Concurrent lifestyle modifications, such as the embrace of Western food patterns abundant in refined carbs and unbalanced fats, together with sedentary behaviors, aggravate gut dysbiosis and influence the distinct metabolic characteristics of lean MASLD in India. The unique pathophysiological pathways present in lean MASLD require customized diagnostic and treatment strategies. Microbiome-targeted therapies, including probiotics, prebiotics, and Fecal Microbiota Transplantation, exhibit significant potential, especially due to their proven effectiveness in non-obese individuals. These solutions provide a tailored approach for addressing MASLD in a demographic where conventional weight-loss therapies may be less effective. Future research should emphasize larger, methodologically designed studies targeting the Indian population to clarify specific microbial signatures, their functional aspects, and the long-term effectiveness of microbiome-modulating strategies in the prevention and treatment of lean MASLD.
